# The Availability of the α7-Nicotinic Acetylcholine Receptor in Early Identification of Vulnerable Atherosclerotic Plaques: A Study Using a Novel ^18^F-Label Radioligand PET

**DOI:** 10.3389/fbioe.2021.640037

**Published:** 2021-03-12

**Authors:** Dawei Wang, Yong Yao, Shuxia Wang, Huabei Zhang, Zuo-Xiang He

**Affiliations:** ^1^State Key Laboratory of Cardiovascular Disease, Department of Nuclear Medicine, Fu Wai Hospital, National Center for Cardiovascular Diseases, Chinese Academy of Medical Sciences and Peking Union Medical College, Beijing, China; ^2^Key Laboratory of Radiopharmaceuticals of Ministry of Education, College of Chemistry, Beijing Normal University, Beijing, China; ^3^Department of Nuclear Medicine, Beijing Tsinghua Changgung Hospital, Tsinghua University, Beijing, China

**Keywords:** vulnerable atherosclerotic plaques, ApoE-/-mice, α7-nicotinic acetylcholine receptor, 18 F-labeled, PET imaging agent

## Abstract

**Background:** It has been confirmed that the α7-nicotinic acetylcholine receptor (α7nAChR) is an important target for identifying vulnerable atherosclerotic plaques. Previously, we successfully designed and synthesized a series of ^18^F-labeled PET molecular probes targeting α7nAChR, which are mainly used in the diagnosis of Alzheimer's disease. Based on the characteristics of α7nAChR in blood vessels, we have firstly screened for a suitable novel ^18^F-labeled PET molecular probe ([^18^F]YLF-DW), with high selectivity for α7nAChR over α4β2nAChR and a good effect for the imaging of atherosclerotic animal models, to effectively identify vulnerable atherosclerotic plaques at an early stage. Meanwhile, we compared it with the “gold standard” pathological examination of atherosclerosis, to verify the reliability of [^18^F]YLF-DW in early diagnosis of atherosclerosis.

**Methods:** The vulnerable atherosclerotic plaques model of ApoE-/-mice were successfully established. Then based on the methods of 3D-QSAR and molecular docking, we designed oxazolo[4,5-b] pyridines and fluorenone compounds, which are targeted at α7nAChR. Through further screening, a novel alpha7 nicotinic acetylcholine receptor radioligand ([^18^F]YLF-DW) was synthesized and automatically ^18^F-labeled using a Stynthra RNplus module. Subsequently, we employed [^18^F]YLF-DW for the targeting of α7nAChR in atherosclerotic plaques and control group, using a micro-PET/CT respectively. After imaging, the mice were sacrificed by air embolism and the carotid arteries taken out for making circular sections. The paraffin embedded specimens were sectioned with 5 μm thickness and stained with oil red. After staining, immunohistochemistry experiment was carried out to verify the effect of micro-PET/CT imaging.

**Results:** The micro-PET/CT imaging successfully identified the vulnerable atherosclerotic plaques in the carotid arteries of ApoE-/-mice; whereas, no signal was observed in normal control mice. In addition, compared with the traditional imaging agent [^18^F]FDG, [^18^F]YLF-DW had a significant effect on the early plaques imaging of carotid atherosclerosis. The results of oil red staining and immunohistochemistry also showed early formations of carotid plaques in ApoE-/-mice and provided pathological bases for the evaluation of imaging effect.

**Conclusion:** We innovated to apply the novel molecular probe ([^18^F]YLF-DW) to the identification of vulnerable atherosclerotic plaques in carotid arteries, to detect atherosclerosis early inflammatory response and provide powerful input for the early diagnosis of atherosclerotic lesions, which may play an early warning role in cardiovascular acute events.

## Introduction

The harmfulness of cardiovascular diseases (CVDs) to human is self-evident. According to a report from the World Health Organization's Global Burden of Disease Assessment, cardiovascular diseases become the main cause of global human mortality (World Health Organization, 2017). Many risk factors of CVDs are related to atherosclerosis (Cole and Kramer, 2015). Despite tremendous advances in the treatment of CVDs, over the past few decades, the incidence of atherosclerosis and its associated complications remain high (Lloyd-Jones et al., 2009; Writing Group et al., 2016). It was also found that plaque inflammation plays an important role in the progression of atherosclerosis (Libby, 2002). Although some features of atherosclerotic plaques can be observed by imaging techniques, such as CT, MRI and intravascular ultrasound (IVUS), others are at the molecular level and beyond the resolution of most imaging techniques (Lovett et al., 2005; Saam et al., 2007; Huibers et al., 2015; Tarkin et al., 2016). Due to these limitations, the early identification of vulnerable atherosclerotic plaques is still an issue. Positron emission computed tomography (PET) has advantages over the above imaging techniques through multimodal-imaging contained anatomical and molecule functional images, thus it is able to visualize molecular mechanisms with high sensitivity (Tarkin et al., 2014; Evans et al., 2016; Chen et al., 2020). PET uses high affinity drugs (ligands) labeled with medical radionuclides. In addition to its high sensitivity, another advantage of PET is that its radioactivity *in vivo* or target tissue is absolutely quantitative. For example, the standard uptake value (SUV) or target to background ratio (TBR), is often used to quantify the uptake of PET tracer in arterial angiography(Bucerius et al., 2016). The key of PET imaging is to identify a suitable tracer for the appropriate target in vascular plaques.

Nicotinic acetylcholine receptors (nAChRs) are pentameric ligand-gated cation channels, which are mainly expressed in the central and peripheral nervous systems diseases (Picciotto et al., 2000; Aharonson et al., 2020). Recently, it become evident that nAChRs are also extensively expressed on non-neuronal cells, such as lung epithelial, endothelial, and immune cells (Wessler et al., 1999; Conti-Fine et al., 2000; Brüggmann et al., 2002; Heeschen et al., 2002; Moccia et al., 2004). Multiple subtypes of nAChRs exist, differing by the type and arrangement of five subunits surrounding an ion channel pore, and which include α (i.e., α2-α10) and β (i.e., β 2-β 4) subunits (Dani and Bertrand, 2007). The nAChR structure affects the pharmacology, cation selectivity, and desensitization kinetics of the receptors. Among the subtypes, the α7nAChRs are composed of five identical α7 subunits. Many studies indicated that alpha7 nicotinic acet-ylcholine receptor (α7nAChR) had the promotion in tumor-induction, furthermore, the lower α7nAChR expression indicated less chemotherapeutic drugs resistance (Hajiasgharzadeh et al., 2020; Zhang et al., 2020; Zheng et al., 2020). Meanwhile, nAChRs also play an important role in the pathophysiology of inflammation in atherosclerotic plaque (Zhao, 2016; Fujii et al., 2017). Among the subtypes, the α7nAChRs are composed of five identical α7 subunits, which appear to be involved in the occurrence and development of atherosclerosis and are becoming one of important targets for early identification of vulnerable atherosclerotic plaque (Sine, 2002; Wilund et al., 2009; Hashimoto et al., 2014; Johansson et al., 2014; Chen et al., 2016).

**Figure 1:**
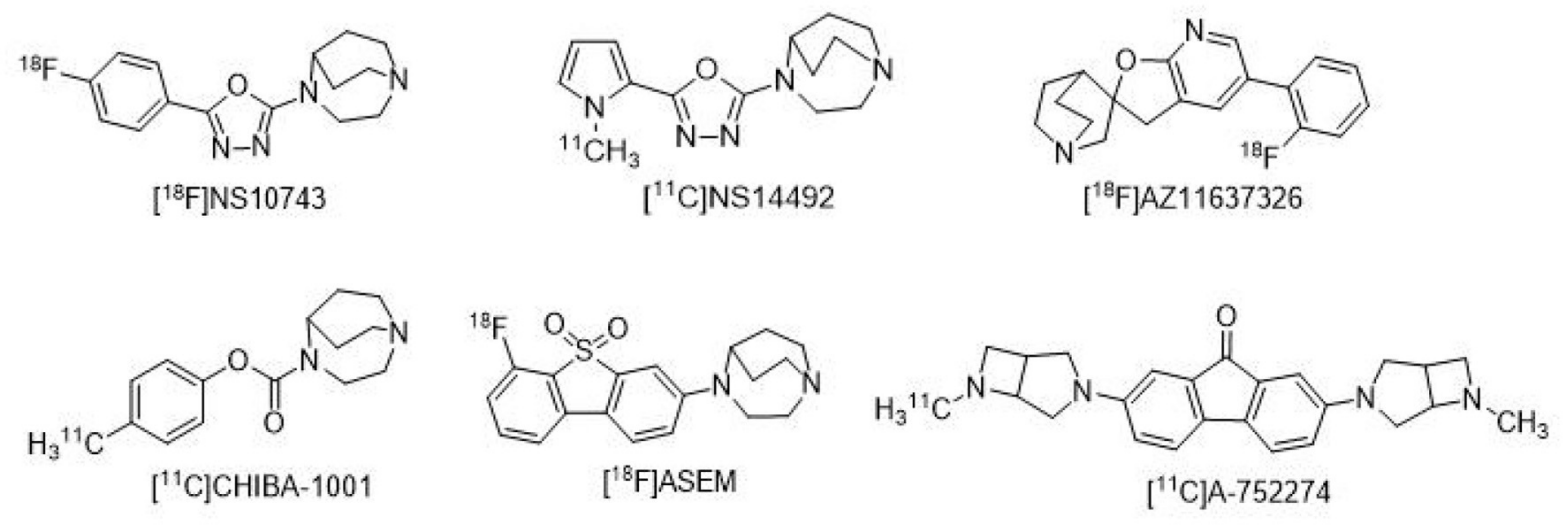
Some representative α7nAChR radioligands.

Previously published reviews have described the development of radiotracers suitable for the *in vivo* imaging of α7nAChR (Toyohara et al., 2010; Brust et al., 2012; Bauwens et al., 2015; Kassenbrock et al., 2016). In the past decade, many researchers have been working on the development of a clinically available imaging agent for an ideal target to non-target ratio through safe and efficient drug delivery (Chen et al., 2017; Feng et al., 2017; Wang J. et al., 2018; Ding et al., 2019; Qiu et al., 2020). About 20 kinds of F-18 or C-11 labeled α7nAChR radioactive PET tracers have been developed. Contributed to the specially binding to α7nAChR, several α7nAChR radioligand had been successfully applied in Alzheimer's Disease, but there have not yet successfully reported in detection of unstable atherosclerosis *in vivo* (Gao et al., 2013; Wang S. et al., 2018). The structure of some representative reported α7nAChR PET tracers is shown in Figure 1 (Boswijk et al., 2017).

All above-mentioned tracers have been mainly studied for neuroimaging applications. In addition, α7nAChR appears to be involved in multiple atherogenic processes *in vitro* (Wang et al., 2016). The ideal features for an imaging PET tracer of vasculature receptors expression differ from those required for imaging of brain receptors. For instance, a correct increase in radioligands hydrophilicity will not pass through the blood-brain barrier and will not allow interference with the signal in the brain; thus, enhancing the signal on the atherosclerotic plaques and increasing the specificity. For these reasons, α7nAChR as a new potential drug target in atherosclerosis diagnosis and treatment, have received an increasing interest in the fields of science and medicine.

Our research group has previously designed and synthesized a series of radioligands for α7nAChR targeting in the brain, with K_i_ values ranging from 0.005 to 450 nM, which exceeded the affinity of similar molecules reported in the literature. Based on the previous experiments and related literature (Rotering et al., 2014; Xue et al., 2016; Boswijk et al., 2017; Teodoro et al., 2018), we first used the novel designed and synthesized high-sensitivity molecular probe ([^18^F]YLF-DW) to identify vulnerable plaques and detect atherosclerosis early inflammatory response, to provide powerful help for the early diagnosis of atherosclerotic lesions and to truly detect early warnings of cardiovascular acute events.

## Materials and Methods

### Design and Screening of Ligands Based on α7nAChR

**Figure 2:**
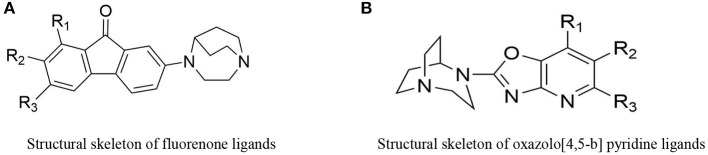
**(A)** Structural skeleton of fluorenone ligands. **(B)** Structural skeleton of oxazolo[4,5-b] pyridine ligands.

**Figure 3:**
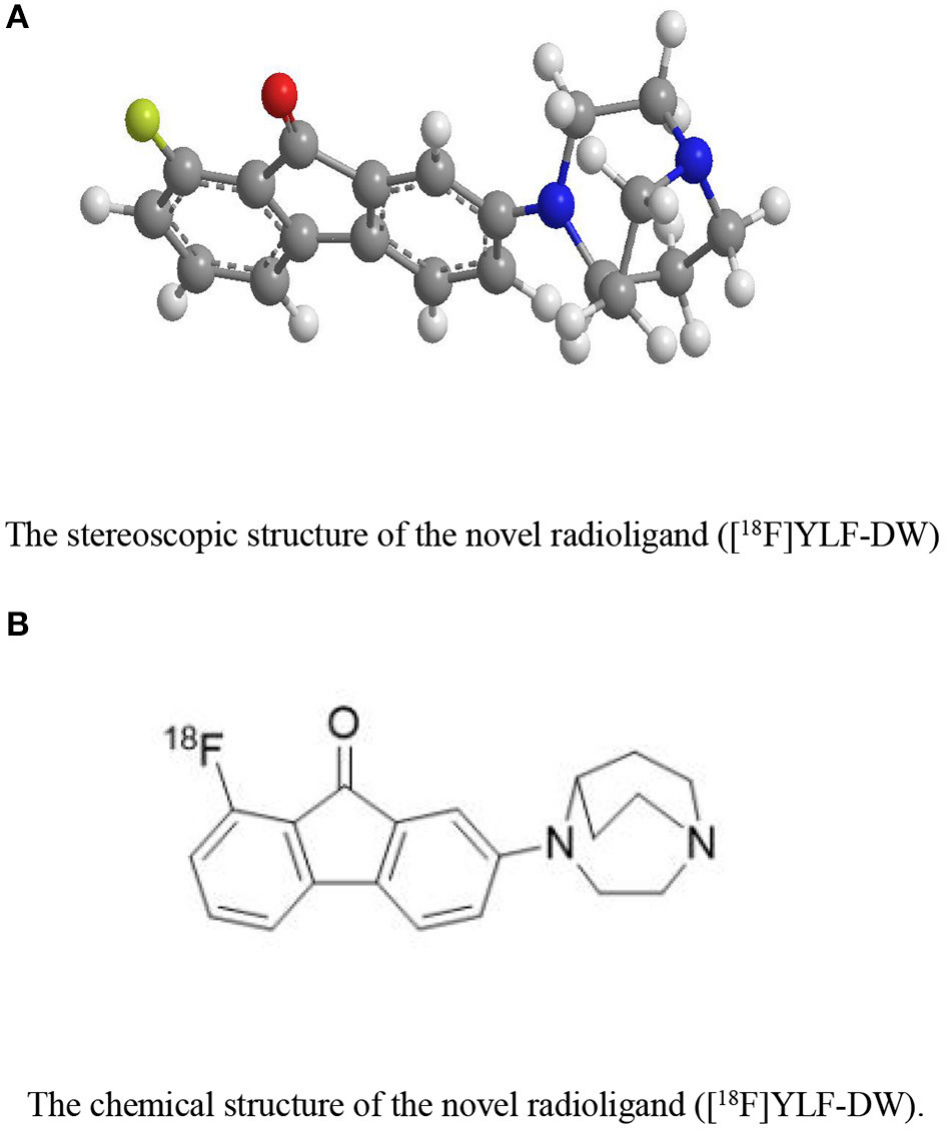
**(A)** The stereoscopic structure of the novel radioligand ([^18^F]YLF-DW). **(B)** The chemical structure of the novel radioligand ([^18^F]YLF-DW).

The ligands based on α7nAChR can be mainly divided into two types: fluorenone derivatives and oxazolo [4,5-b]pyridine derivatives (Figure 2), In addition the active α7nAChR agonist is generally composed of three parts: basic segment, aromatic group, and connecting segment. We used the methods of the three-dimensional quantitative structure-activity relationship (3D-QSAR) and molecular docking, in computer-aided drug design (CADD) (supplementary materials for design details), and Finally screened for the suitable novel ligand ([^18^F]YLF-DW) for atherosclerotic vulnerable plaques (The structure of [^18^F]YLF-DW is shown in Figure 3).

### Radiochemistry of [^18^F]YLF-DW

**Figure 4:**
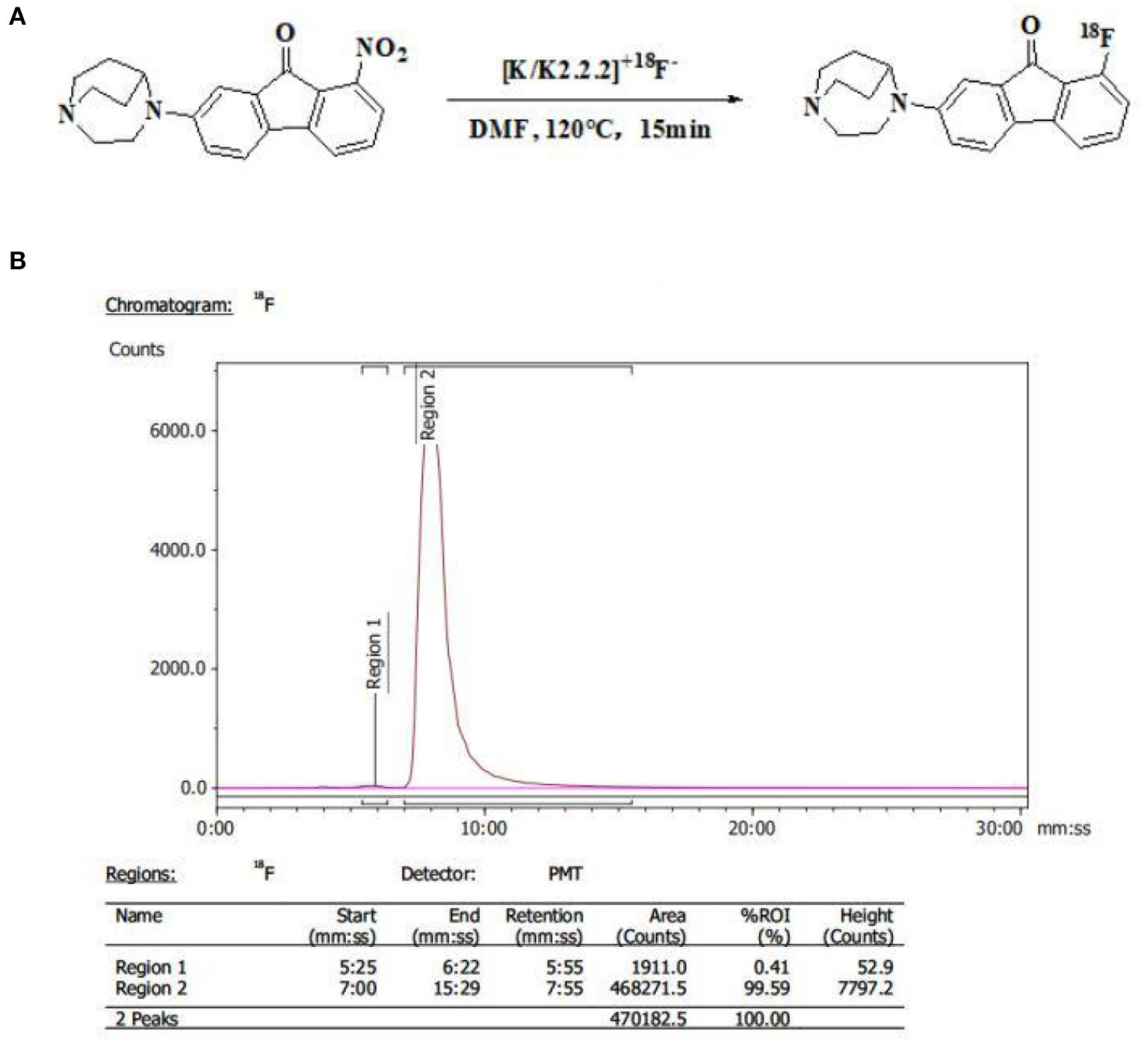
**(A)** Synthetic scheme of [^18^F]YLF-DW. **(B)** Radio-high-performance liquid chromatography profile of [^18^F]YLF-DW.

[^18^F]YLF-DW was labeled using a Synthra RNplus AAQQ67 module (Synthra GmbH, Hamburg, Germany). First, the ^18^F-produced by the accelerator (Cyclone 18 twin, IBA, Belgium) and adsorbed in the QMA pillars, was eluted by 1 ml eluent (15 mg K.2.2.2., 1.5 mg K_2_CO_3_, 0.6 mg acetonitrile, 0.4 mL H_2_O). Second, the precursor (3 mg) and DMSO (0.5 mL) were added to the reaction vial and the reaction was carried out at 120°C for 15 min; subsequently, the reactant was diluted and separated and the diluent passed through a C18 column to remove impurity. Finally, the C18 column was eluted with ethyl alcohol (1 mL); thereafter, the leachate was diluted by normal saline (10 mL) to acquire the ^18^F-labeled Radioligand injection. The total time was ~30 min with a radiochemical yield of 10–15% and a radiochemical purity >98% (Figure 4).

### Animal Model Preparation

ApoE-/-mice (*n* = 6, weight 25–33 g) and normal mice (*n* = 6, weight 25–33g) were purchased from Qingzilan Technology Co., Ltd (Nanjing, China). After 1 week of feeding with common feed, the selected ApoE-/-mice were fed with western diet, containing 21% fat and 0.15% cholesterol content, with a high-fat/high-cholesterol content for 9–10 weeks and subjected to micro-PET/CT imaging. The tissues were used for red staining and immunohistochemistry experiments. The normal mice served as controls for micro-PET/CT imaging and their tissues for immunohistochemistry. All mice were kept in a temperature-controlled environment with a 12 h light/dark cycle with free access to food and water. Animal care and experimental procedures were conducted in compliance with the Helsinki Declaration and approved by Fuwai hospital animal care committee.

### Blocking Experiments

To further determine the specific binding of [^18^F]YLF-DW to α7nAChR over other similar receptors, blocking experiments were carried out by pre-subcutaneous injection of blocking agents. α4β2nAChR, a heteromeric nAChR subtype widely expressed and 5-HT3, a receptor sharing high sequence homology with α7nAChR, were the most likely receptors to cross binding with α7nAChR ligands. We used the brain tissues of CD-1 mice to do the blocking experiment. Cytisine (1 mg/kg, a selective partial agonist of α4β2nAChR) and ondansetron (2 mg/kg, a selective antagonist of 5-HT3) were pre-injected 5 and 10 min prior to the intravenous injection of [^18^F]YLF-DW, respectively. The CD-1 mice were sacrificed at 60 min after the [^18^F]YLF-DW injection, and the brain tissues were harvested.

### Micro-PET/CT Imaging

When the animal models were 10 and 11 weeks old, the micro-PET/CT imaging were conducted. An Inveon small animal PET/CT scanner (Siemens,Germany) was used to perform the scans. A total volume of 5.56 MBq (150 μCi) of [^18^F]YLF-DW was injected into six ApoE-/-mice and six normal mice by the tail veins, and the animal models were anesthetized with 2% isoflurane through inhalation. We performed PET/CT dynamic scanning. After screening, the acquisition was performed at 1 h after tail vein injections. The micro-PET/CT was initially performed with the following parameters: energy peak, 511 keV; window width, 3.5 E0 SUV-bw; resolution, 0.775 mm/pixel; and matrix, 128 × 128. Each mouse required a 15-min acquisition and the computed tomography (CT) was performed after PET scanning in the same bed position using the following parameters: frame resolution, 256 × 512; tube voltage, 80 kVp; current, 0.5 mA; and exposure time, 500 ms/frame.

The images were reconstructed by two-dimensional-ordered subsets expectation maximization. The three-dimensional regions of interest (ROIs) were superimposed on the high uptake region in carotid arteries of ApoE-/-mice. The tissue density was presumed as 1 g/ml, and % ID/g was calculated based on the dose injected into the mice.

### *In vivo* Biodistribution Studies in Mice

After dynamically micro-PET/CT imaging for 1 h. The primary data were reconstructed by OSEM 3D strategy on MicroQ software through 4 times iteration. The region of interest of the heart, liver, spleen, lung, kidney, aorta, muscle, and bone tissues were outlined on the CT background were with the post-reconstruction Inversion Reconstruction Workstation. The % ID/g value at each period were automatically obtained.

### Red Staining Experiment and Immunohistochemistry Experiment

Six carotid arteries from ApoE-/-mice and normal mice, that underwent *in-vivo* micro-PET/CT imaging, were selected for oil red staining and immunohistochemistry experiments.

#### Oil Red Staining Experiment

First, the frozen sections of the carotid artery were rewarmed and dried, fixed in the fixative (G1101, Servicebio) for 15 min, washed with running water and dried. Then, the slices were dipped into the oil red dye solution (G1016, Servicebio) for 8–10 min (cover to avoid light), washed with distilled water, slightly differentiated with 75% alcohol and washed with distilled water. Afterward, the slices were dyed with hematoxylin solution (G1004, Servicebio) for 3–5 min, washed with tap water, differentiated with differentiation solution, washed with tap water, returned to blue with returned blue solution (G1005-4, Servicebio) and washed with running water. After the film is sealed with glycerin gelatin (G1402, Servicebio), the dyed slices were examined under microscope and the images were collected and analyzed.

#### Immunohistochemistry Experiment

The slides were successively put into xylene I, II, and III for 15 min each, anhydrous ethanol I and II for 5 min each, 85% ethanol for 5 min, 75% ethanol for 5 min, and washed with distilled water. Then, the tissue sections were placed in the repair box filled with citric acid antigen repair buffer (pH 6.0) and repaired in the microwave oven. The reaction mixtures were heated to boil within 8 min, after boiled maintained heating for 8 min and then kept heating for 7 min under medium-low power. In this process, excessive evaporation of the buffer should be prevented, and the chips should not dry. After natural cooling, the slides were placed in PBS (pH 7.4) and shaken on the bleaching shaker for 3 times, each time for 5 min. The slides were then put into 3% hydrogen peroxide solution, incubated at room temperature in the dark for 25 min, placed in PBS (pH 7.4) and washed on the decolorizing shaking table for 3 times, each time for 5 min. A solution of 3% BSA was dripped into the histochemical circle to cover the tissue uniformly and the tissues were sealed for 30 min at room temperature These steps were followed by a gentle shaking off the sealing solution, PBS drops were added on the slides to prepare for the first antibody (Alpha7 antibody, abcam, Ab216485) in a certain proportion, and incubated flat in the wet box at 4°C for incubation overnight (add a small amount of water in the wet box to prevent the antibody solution from evaporating). After incubation, the slides were washed in PBS (pH 7.4) and shaken for 3 times, each time for 5 min. After, the slides were slightly dried, labeled HRP corresponding to the first antibody species were dripped into the circles and incubated at room temperature for 50 min. After this incubation step, the slides were washed in PBS (pH 7.4) for decolorization on a shaker for 3 times, each time for 5 min and slightly dried. DAB (G1211, Servicebio) was used to develop color, which was controlled under the microscope. When the brown yellow color developed (positivity), the slides were washed with tap water to stop the color further developing. For the hyperchromatic nucleus, hematoxylin was re-dyed for ~3 min, washed with tap water, differentiated with hematoxylin differentiation solution for several seconds, washed with tap water, turned blue with hematoxylin return solution and washed with running water. Finally, the slides were treated with 75% alcohol for 5 min, 85% alcohol for 5 min, anhydrous ethanol for 15 min, anhydrous ethanol for 5 min, xylene for 5 min to dehydrate and render transparent, slightly dried, and sealed with neutral gum. Then, the images were collected and analyzed.

### Statistical Analysis

All the reported data were expressed as mean and SD. *P*-values < 0.05 were considered as statistically significant.

## Results

### *In vivo* Biodistribution Studies

**Figure 5:**
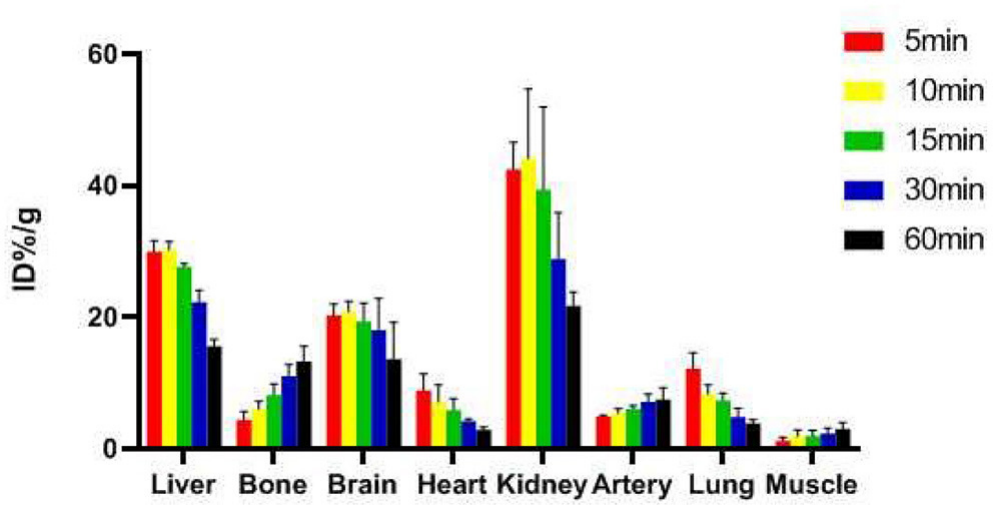
Biological distribution of mice.

In the previous experiment, we have measured the K_i_ value of the novel molecular probe (K_i_ = 2.98 ± 1.41 nM) and also made blocking experiments to prove that the ligand is specific binding to α7nAChR^33^. Evaluated the biodistribution of the tracer in normal mice (Figure 5). The highest accumulation of radioactivity was observed in the kidney, liver and brain, with a peak uptake at 10 min after injection before decreasing. The lowest uptake was found in the muscle and bone. The distribution of the novel radioligand in the artery was consistent with the distribution of α7nAChRs in the arterial vessels, which uptake increased over time, with peak uptake at 60 min.

### Blocking Experiment Studies

Cytisine is a selective agonist of α4β2nAChR, and Ondanstron is a selective antagonist of 5-hydroxytryptamine receptor. we have done relevant selection experiments. there is no significant difference in the uptake of this novel radioligand between the experimental group and the control group. [^18^F]YLF-DW(Ki = 2.98 ± 1.41 nM) exhibited high initial brain uptake (11.60 ± 0.14%ID/g at 15 min post-injection), brain/blood value (9.57 at 30 min post-injection), specific labeling of a7-nAChRs and fast clearance from Kunming mouse brains, which demonstrated that [^18^F]YLF-DW has good selectivity to α7 nAChR (Wang S. et al., 2018).

### Micro-PET/CT Imaging Studies Between Nomal and ApoE-/-Mice

**Figure 6:**
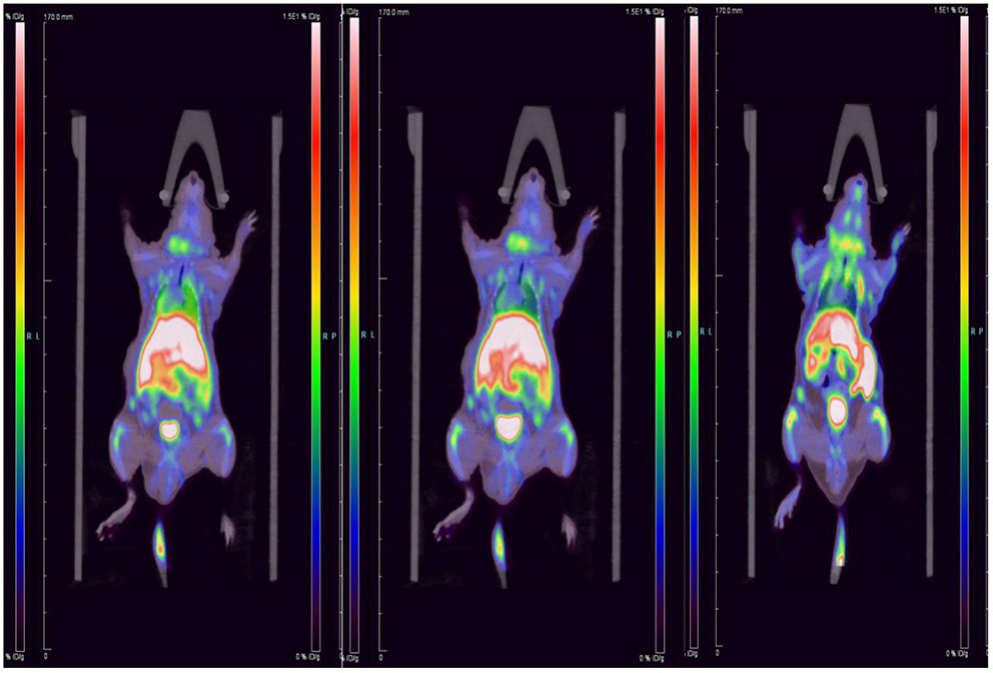
Dynamic scanning: 5 min imaging on the left, 15 min imaging in the middle, 30 min imaging on the right.

**Figure 7:**
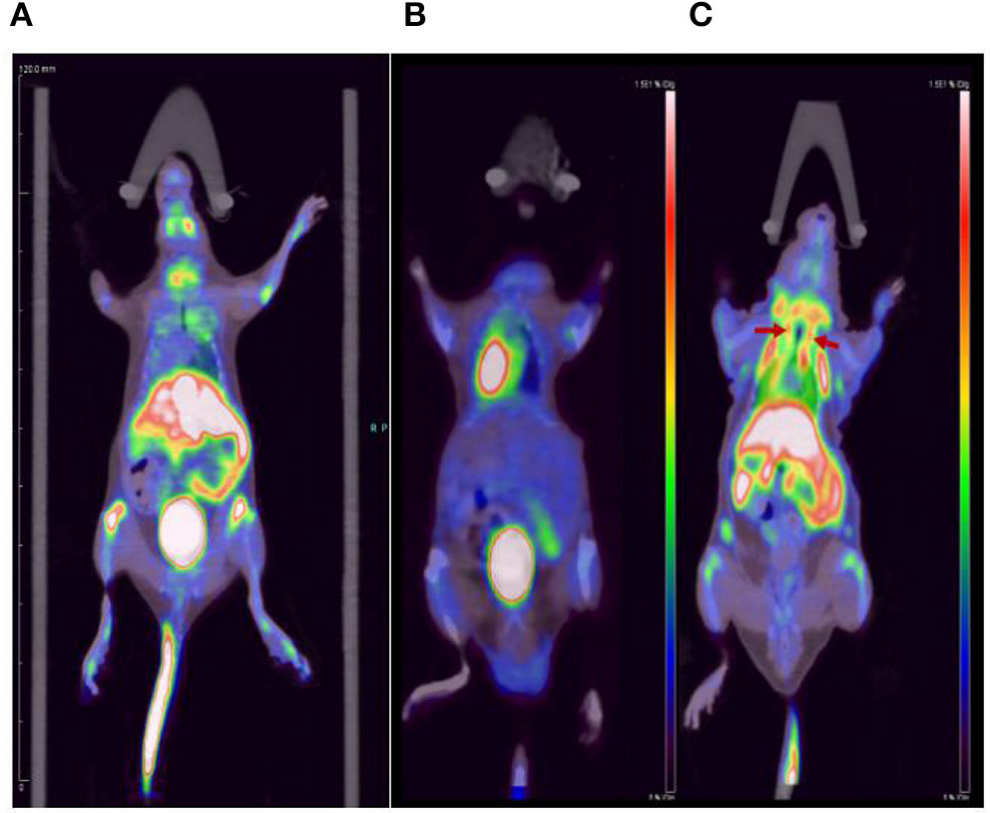
Comparison of **(A)** the normal mouse, **(B)** [^18^F]FDG carotid plaques imaging, and **(C)** [^18^F]YLF-DW carotid plaques imaging.

**Figure 8:**
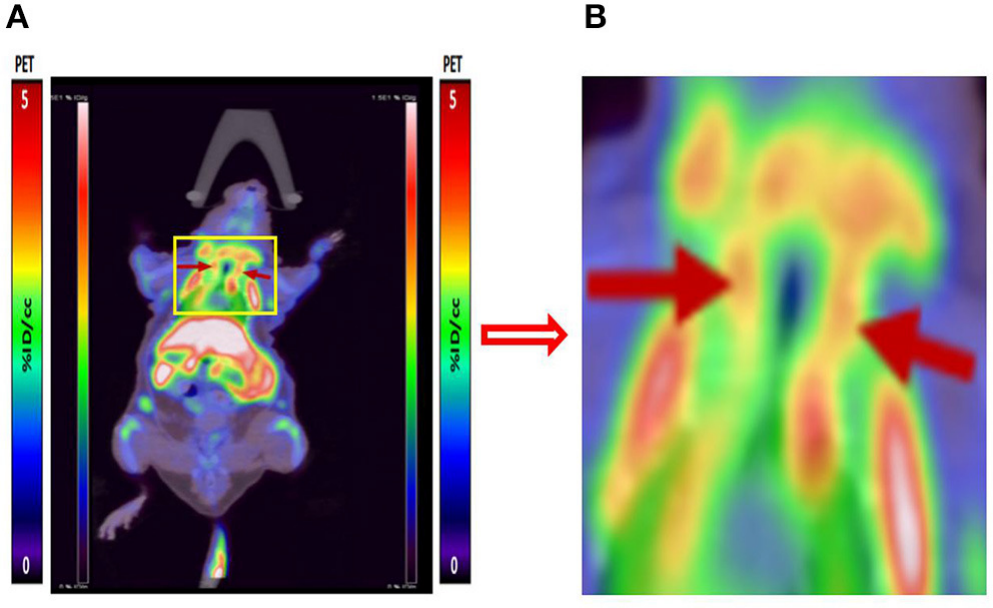
**(A)** [^18^F]YLF-DW carotid plaques imaging of ApoE-/-mouse. **(B)** Local enlarged view of carotid artery imaging.

We performed micro-PET/CT dynamic scanning on the animal models. After comparative analysis, it is found that the carotid artery imaging effect is better contrast byound other section after 1 h of scanning, which can be used for later clinical guidance. We also attached 5, 15, and 30 min of scanned images to the paper for comparison (Figure 6). [^18^F]YLF-DW images one of the ApoE-/-mouse models and normal mouse models are shown in Figure 7. Compared with the normal mouse(a) and the traditional [^18^F]FDG of the ApoE-/-mouse(b), [^18^F]YLF-DW was obvious in the carotid artery of the ApoE-/-mouse(c). (Red arrows in the right figure below). This suggests that [^18^F]YLF-DW is better than [^18^F]FDG in the early detection of carotid plaques. In addition, Figure 8 shows [^18^F]YLF-DW carotid plaque imaging of the ApoE-/-mouse alone (The carotid plaques was obviously imaged).

### Oil Red Staining

**Figure 9:**
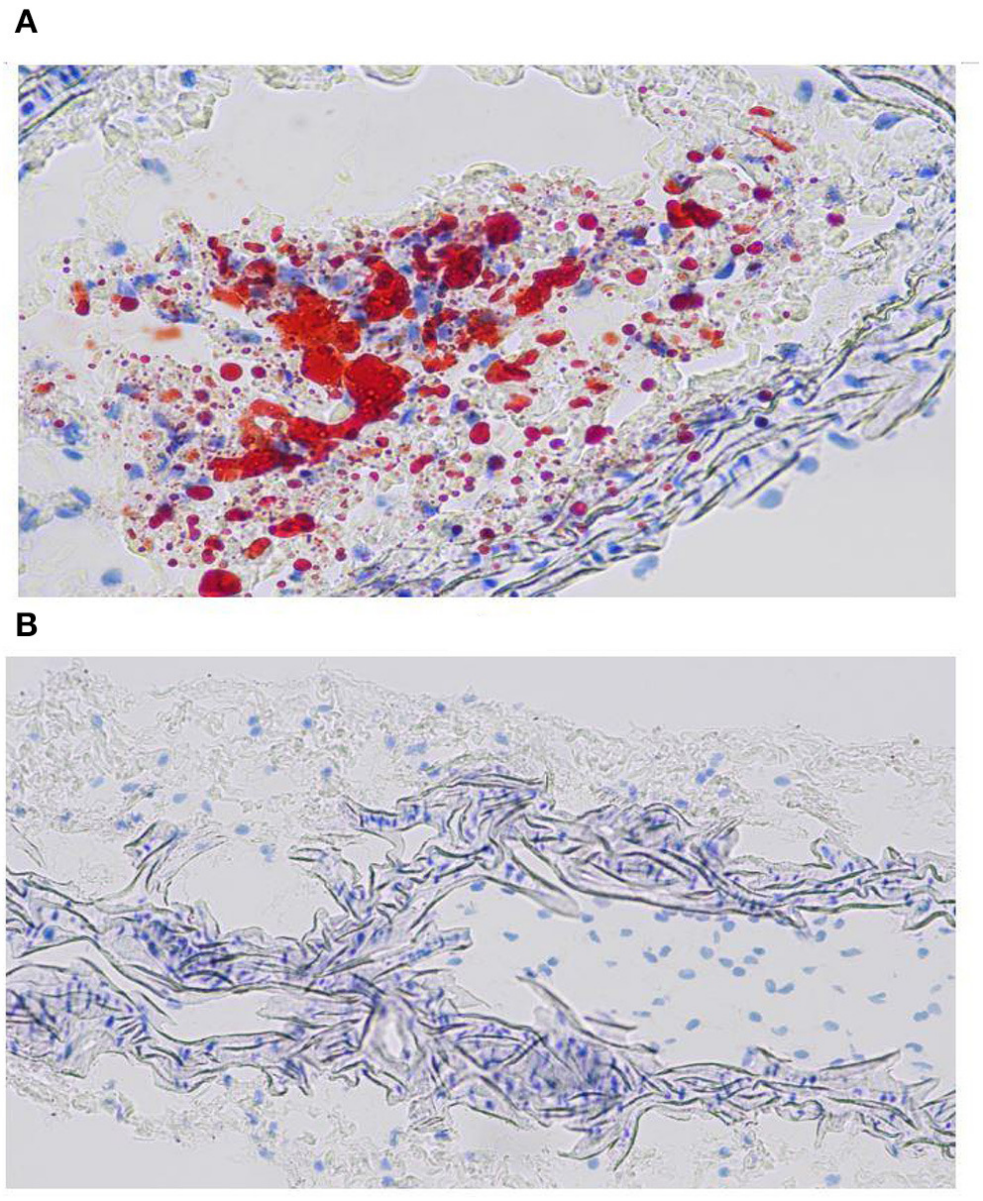
Oil red staining chromatogram of ApoE-/-mice **(A)** and normal mice **(B)**.

Under the light microscope, the layers of the carotid artery were arranged in order. Oil red O staining of ApoE-/-mice and normal mice showed that the area of the atherosclerotic plaques was bright red in fat, blue in nucleus and colorless in stroma. The structure is clear, the cell morphology is good and there are few wrinkles and vacuoles (Figure 9). The section clearly showed the formation of extensive and typical atherosclerotic plaques in the carotid arteries of ApoE-/- mouse, which reflects the presence of lipid content in the atherosclerotic plaques in ApoE-/- mouse and provide the basis for the early diagnosis of atherosclerosis.

### Immunohistochemistry

**Figure 10:**
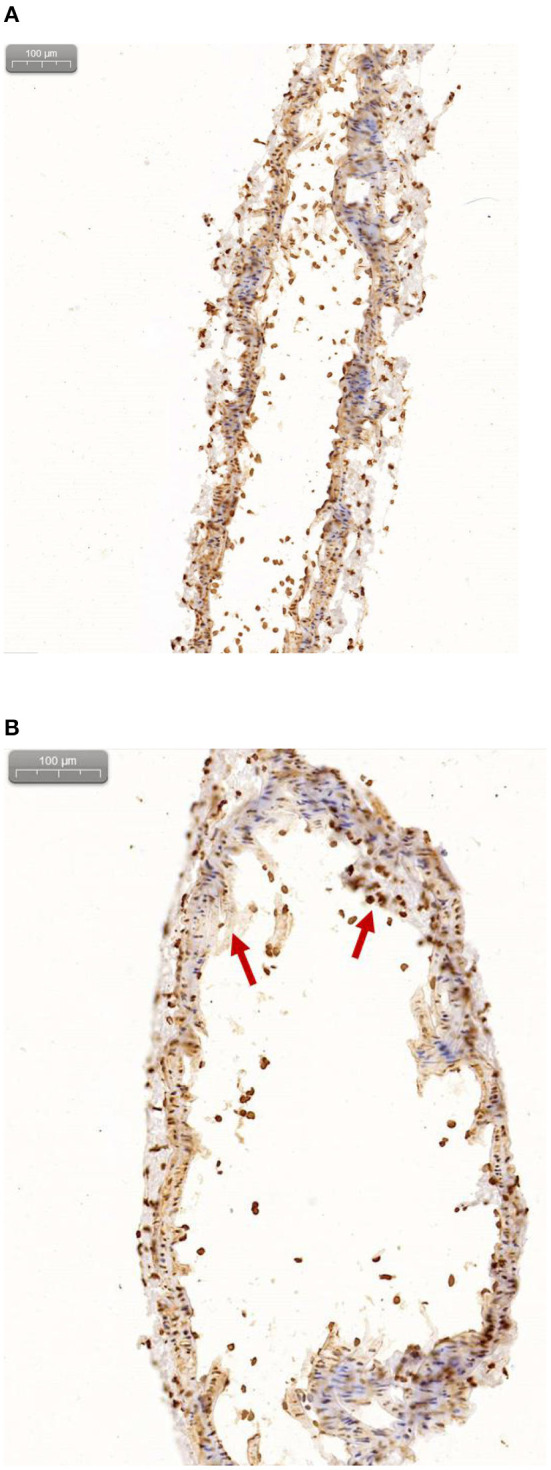
**(A)** Immunohistochemical results of normal mice. **(B)** Immunohistochemical results of ApoE-/-mice.

Figure 10 shows the immunohistochemistry of the carotid artery in control mice (a), and the immunohistochemistry of the carotid artery in the ApoE-/-mice (b) (Red arrows indicates plaques formation). Hematoxylin stained cell nucleus is shown in blue and the positive DAB staining is brownish yellow. The lesions with accumulated radioactivity agreed with the anatomical structure of the plaques, which confirms the imaging effect of micro-PET/CT and further explains the early formation of vulnerable plaques.

## Discussion

Until now, the prediction of vulnerable atherosclerotic plaques, that are thrombotic triggers, tightly linked to inflammation, was challenging. Although [^18^F]FDG has been used for atherosclerotic inflammation imaging for several years, but the low cell specificity of [^18^F]FDG restrict the use of FDG in atherosclerosis (Bucerius et al., 2016). As it has been proved that α7-nicotinic acetylcholine receptor (α7nAChR) in blood vessels is closely related to atherosclerotic plaques, imaging α7nAChR would provide a unique tool to identify atherosclerotic plaques that are prone to rupture. Therefore, it becomes very important to study appropriate PET molecular probes that can specifically bind to α7nAChR in vascular plaques.

In the present study, we established plaque models of abdominal and carotid arteries in ApoE-/-mice. We found that the abdominal artery of the ApoE-/-mice was not easy to model, and that early plaques could not be observed by pathology. On the contrary, the mouse carotid artery plaques is easy to model, which was confirmed by immunohistochemistry. Therefore, we chose the mouse carotid artery as the subject of our study. Meanwhile, the formation of vulnerable atherosclerotic plaques is a complex process involving inflammation, microcalcification and apoptosis. Therefore, the evaluation of the vulnerability of atherosclerotic plaques by PET/CT imaging, with an appropriate molecular probe for α7nAChR in blood vessels, also has an important clinical application value.

In the past work, we mainly used manual labeling. In this study, we used automatic modular instrument to shorten the synthesis time, improve the purification rate and labeling rate (total synthesis time is about 15 min, radiochemical yield is 10–15%, radiochemical purity is over 99.5%). Dynamic scanning method can screen out the best imaging conditions, which is helpful to guide the optimization of subsequent probes.

This study shows the potential of the novel molecular probe for early diagnosis of vulnerable atherosclerotic plaques. Compared with the previous diagnosis of plaques, the imaging system is more sensitive to their detection, and may present a higher plaque signals. Therefore, PET/CT imaging can be used to evaluate the effect of α7nAChR targeting strategies in the diagnosis of atherosclerosis.

## Conclusion

In the current study, a novel radioligand micro-PET/CT imaging successfully explored the atherosclerotic plaques in mouse models [the acute toxicity studies and blocking studies of this probe have been finished (Wang S. et al., 2018)]. The results supported the feasibility of the appropriate molecular probe as a α7-targeting tracer to detect the inflammation in atherosclerotic plaques, which holds the potential to assess the vulnerability of atherosclerotic plaques rupture. Therefore, the molecule probe has potential to be an appropriate α7-nAChR-targeted imaging radiotracer for early plaques diagnosis, and further studies on these radioligand series are ongoing.

## Data Availability Statement

The original contributions presented in the study are included in the article/supplementary material, further inquiries can be directed to the corresponding authors.

## Ethics Statement

All protocols requiring the use of ApoE-/-mice were approved by the Animal Care Committee of Fu Wai Hospital.

## Author Contributions

Z-XH and HZ designed experiments. DW and SW performed experiments. DW and YY analyzed data. DW wrote the manuscript. All authors contributed to the article and approved the submitted version.

## Conflict of Interest

The authors declare that the research was conducted in the absence of any commercial or financial relationships that could be construed as a potential conflict of interest.

## Abbreviations

α7: alpha 7 nicotinic acetylcholine receptor
PET: positron emission tomography
CT: computed tomography
CVD: cardiovascular disease
DMF: dimethylformamide
HPLC: high performance liquid chromatography
AD: Alzheimer's disease
K_i_: inhibitory constant
NMR: nuclear magnetic resonance
nM: nanomolar
IVUS: intravascular ultrasound
CADD: computer-aided drug design
3D-QSAR: three-dimensional quantitative structure-activity relationships.
